# The Ketogenic Diet as Adjunctive Strategy in Cancer Treatment—Therapeutic Benefits and Metabolic Risk Using Breast Cancer as an Example

**DOI:** 10.3390/nu18172917

**Published:** 2026-09-06

**Authors:** Jacek Szołtysek, Beata Szymańska, Agnieszka Piwowar

**Affiliations:** 1 Students’ Scientific Society, Department of Toxicology, Faculty of Pharmacy, Wroclaw Medical University, 50-556 Wroclaw, Poland; 2 Department of Toxicology, Faculty of Pharmacy, Wroclaw Medical University, 50-556 Wroclaw, Poland

**Keywords:** ketogenic diet, cancer, breast cancer, metabolic risk, quality of life

## Abstract

Nutritional therapy is an essential component of comprehensive care for cancer patients, playing a vital role in preventing malnutrition, improving treatment tolerance, and maintaining quality of life. In recent years, there has been growing interest in the ketogenic diet as a potential strategy to support cancer therapy. This diet, based on a significant reduction in carbohydrate intake and an increase in fat intake, leads to a state of ketosis that mimics the metabolic changes that occur during starvation. Consequently, the body utilizes ketone bodies as an alternative source of energy. The potential application of the ketogenic diet in oncology stems from the distinct metabolism of cancer cells, which often exhibit an increased demand for glucose. Although the results of preclinical studies suggest a possible anticancer effect of this dietary intervention, clinical evidence remains limited, and its routine use is not currently recommended as a standard of care in oncology. The aim of this study was to analyze the current literature on the use of the ketogenic diet in oncology, with a particular focus on breast cancer. The study assessed the impact of this intervention on metabolic parameters, body composition, and patients’ mental well-being, and analyzed the potential metabolic risks associated with its use. The study discusses the molecular basis of oncogenesis and metabolic adaptation in cancer cells, the mechanisms of action of the ketogenic diet, and the results of preclinical and clinical studies as well as meta-analyses. Importantly, the current evidence base should be interpreted with caution. Most mechanistic evidence supporting ketogenic diet in breast cancer derives from preclinical models, whereas clinical studies remain limited by small sample sizes, short intervention periods, heterogeneous ketogenic diet protocols, substantial attrition, and differences in concomitant anticancer treatment. Therefore, mechanistic plausibility and promising preclinical findings should not be interpreted as evidence of clinical anticancer efficacy.

## 1. Introduction

Epidemiological data indicate that in terminal cancer patients, up to 20% of deaths are a direct consequence of malnutrition or cachexia [1,2]. Preventing weight loss and maintaining a normal body mass index (BMI) are essential elements of clinical management in oncology patients. Malnutrition is a frequent complication of cancer and is associated with a poorer prognosis, an increased complication rate, and reduced treatment efficacy. Therefore, an appropriately planned nutritional therapy plays a key role in the prevention of malnutrition and supports the treatment process. Following a cancer diagnosis, the rapid implementation of treatment and lifestyle modifications, including diet, is of crucial importance, as these can affect the course of therapy, improve the quality of life, and reduce the risk of premature death. One nutritional approach generating increasing interest in oncology is the ketogenic diet and its modifications. This diet is based on a significant restriction of carbohydrate intake combined with increased fat consumption, which leads to the induction of ketosis and increased production of ketone bodies. In this way, it mimics the metabolic changes that occur during fasting. The ketogenic diet currently has recognized clinical applications, primarily in the treatment of drug-resistant epilepsy in children and adults [3], whereas its potential role as an adjuvant therapy in cancer remains the subject of intense research.

A distinction is made between the classic ketogenic diet (CKD) and its variants: the medium-chain triglyceride (MCT) ketogenic diet, the modified Atkins diet (MAD), the low glycemic index treatment (LGIT), and the modified ketogenic diet (MKD), which is less restrictive regarding carbohydrate intake [4]. CKD and MAD are the most commonly used in clinical trials. Literature data suggest that the ketogenic diet offers potential benefits in drug-resistant epilepsy; however, the available scientific literature is largely based on insufficient research data regarding this, as well as other medical conditions. The main controversy surrounding the broader application of ketogenic diets is the low quality of clinical trials and the limited methodological rigor of the studies [5]. The majority of evidence supporting the beneficial effects of the ketogenic diet is derived from animal model studies. Despite promising results in animal models, the use of the ketogenic diet as a standard adjuvant therapy in oncology patients is still not recommended. A significant barrier to the implementation of the ketogenic diet may be the requirement for strict dietary adherence and close supervision by a physician and dietitian due to the risks associated with nausea, vomiting, and diarrhea, which can paradoxically contribute to the development of malnutrition [4].

In light of current scientific reports and existing uncertainties regarding the efficacy and safety of this intervention, the aim of this study was to conduct a critical literature review and analyze the available scientific evidence in relation to the following research questions: whether the use of the ketogenic diet result in potential changes in body composition and metabolic parameters, does the ketogenic diet improve the mental health of oncology patients (with a particular focus on breast cancer patients), and what metabolic risks are associated with the use of the ketogenic diet in breast cancer patients.

## 2. Methodology

This article was designed as a narrative review to summarize the current evidence regarding the ketogenic diet in oncology, with a particular focus on breast cancer. Because of the limited number of high-quality clinical studies specifically involving breast cancer, evidence from studies on other malignancies was also included to provide a broader understanding of the biological rationale, potential mechanisms, and clinical applications of the ketogenic diet in cancer.

A structured literature search was conducted using the electronic databases PubMed, Scopus, Web of Science, and ScienceDirect. In addition, the reference lists of relevant publications were manually screened, and a supplementary web search was performed to identify additional eligible studies. The search strategy combined Medical Subject Headings (MeSH) and free-text terms using Boolean operators (AND, OR). The following keywords were used individually and in combination: “cancer,” “breast cancer,” “ketogenic diet,” “low-carbohydrate diet,” “high-fat diet,” “malnutrition,” “quality of life,” “body composition,” “metabolic parameters,” “metabolic risk,” “chemotherapy,” and “radiotherapy.” As the available evidence on the ketogenic diet in breast cancer was limited and methodologically heterogeneous, the search was expanded from “breast cancer” to the broader term “cancer” to capture studies providing relevant mechanistic and clinical insights.

Eligible publications included meta-analyses, systematic reviews, randomized controlled trials, clinical studies, and narrative reviews. Priority was given to meta-analyses, systematic reviews, and randomized controlled trials published between 2015 and 2026, while earlier landmark studies on cancer metabolism were included when necessary. Only full-text articles published in peer-reviewed journals were considered. Publications unrelated to the review objectives, conference abstracts without full-text articles, and duplicate records were excluded.

Due to the heterogeneity of study designs, cancer types, ketogenic diet protocols, and reported outcomes, a quantitative synthesis was not feasible. Therefore, the evidence was synthesized qualitatively and organized into thematic sections covering the ketogenic diet and its variants, its biological basis and potential role in oncology, challenges associated with its clinical use, and its application in breast cancer, including biological subtypes, interactions with chemotherapy and radiotherapy, quality of life, body composition, nutritional status, and metabolic outcomes.

This article is a narrative review and did not conduct a PRISMA protocol, formal risk of bias assessment, or quantitative meta-analysis. Furthermore, the absence of a meta-analysis reflects significant heterogeneity in the study population, ketogenic diet protocols, treatment settings, outcome measures, study duration, and concomitant anticancer therapies, but should not be interpreted as evidence of clinical efficacy.

## 3. Ketogenic Diet

The classic ketogenic diet was developed by doctor Russell Wilder in 1921. This dietary model is characterized by high energy content of fats and low energy content of carbohydrates, which induces a metabolic state in the body similar to fasting state [3,4,5,6]. Macronutrient composition in classic ketogenic diet (at a ratio of 4 g of fat to 1 g of carbohydrates and proteins) should consist of 90% energy from fats, 6% energy intake from protein and 4% of energy intake from carbohydrates [3]. Crucial aspect is fat to protein and carbohydrates ratio, which in classical ketogenic diet should be 3–4: 1 (from 3 to 4 g of fat to 1 g of protein and carbohydrates) [6]. Fat intake mainly rely on long-chain triglycerides (LCT) and is realized by including to diet products such as: vegetable oils (olive oil, rapeseed oil, sunflower oil), avocado, nuts (walnuts, brazil nuts), animal fats (lard, bacon, pork neck, beef tallow) dairy products (butter, cheese) and fat sea fish (mackerel, salmon, herrings). Dietary models based on an extremely low intake of carbohydrates are associated with vitamin and mineral deficiencies such as vitamin B1, B6, folic acid, vitamin A, E, K, potassium, calcium, magnesium, iron. Moreover ketogenic diet lacks dietary fiber and linoleic acid therefore it is essential to take care of the microbiota because of low intake of dietary fiber [7].

Glucose is an essential source of energy for the proper development of the nervous system. The ketogenic diet, reduces the availability of glycogen forcing the body to switch to an alternative energy source: ketone bodies [8].

After consuming a carbohydrate-rich meal, insulin levels rise. Insulin stimulates lipogenesis and inhibits lipolysis by inhibiting hormone-sensitive lipase (HSL), which is responsible for releasing free fatty acids into the bloodstream. Insulin regulates lipogenesis and the beta-oxidation of fatty acids. In the liver, insulin activates the enzyme acetyl-CoA carboxylase (ACC), resulting in an increase in malonyl-CoA levels. Malonyl-CoA is an inhibitor of the enzyme carnitine palmitoyltransferase 1 (CPT1). An increase in malonyl-CoA concentration inhibits the β-oxidation of fatty acids by blocking CPT1, which plays a key role in the production of ketone bodies. During a ketogenic diet, this mechanism is reversed. Lower blood glucose levels result in decreased insulin levels and stimulate the secretion of glucagon, cortisol, and catecholamines. Low energy levels activate AMP-activated Protein Kinase (AMPK), which causes the phosphorylation and inactivation of the ACC enzyme, leading to a decrease in malonyl-CoA. A deficiency of malonyl-CoA enables the beta-oxidation of fatty acids [9]. Glucose deficiency causes an increase in blood glucagon levels, which, by influencing signaling pathways, inhibits glycolysis and stimulates gluconeogenesis to supply glucose to the brain. For glucose synthesis to proceed, oxaloacetate (OAA) is converted to phosphoenolpyruvate (PEP). This results in the redirection of a significant portion of OAA from the Krebs cycle to the gluconeogenesis pathway. Excess acetyl-CoA strongly activates pyruvate carboxylase, which converts pyruvate to OAA, but this newly formed OAA is immediately consumed in the intensified gluconeogenesis. The concentration of OAA in the mitochondrial matrix remains too low for acetyl-CoA to enter the Krebs cycle. Consequently, acetyl-CoA is redirected to the ketone body synthesis pathway [10].

**Figure 1:**
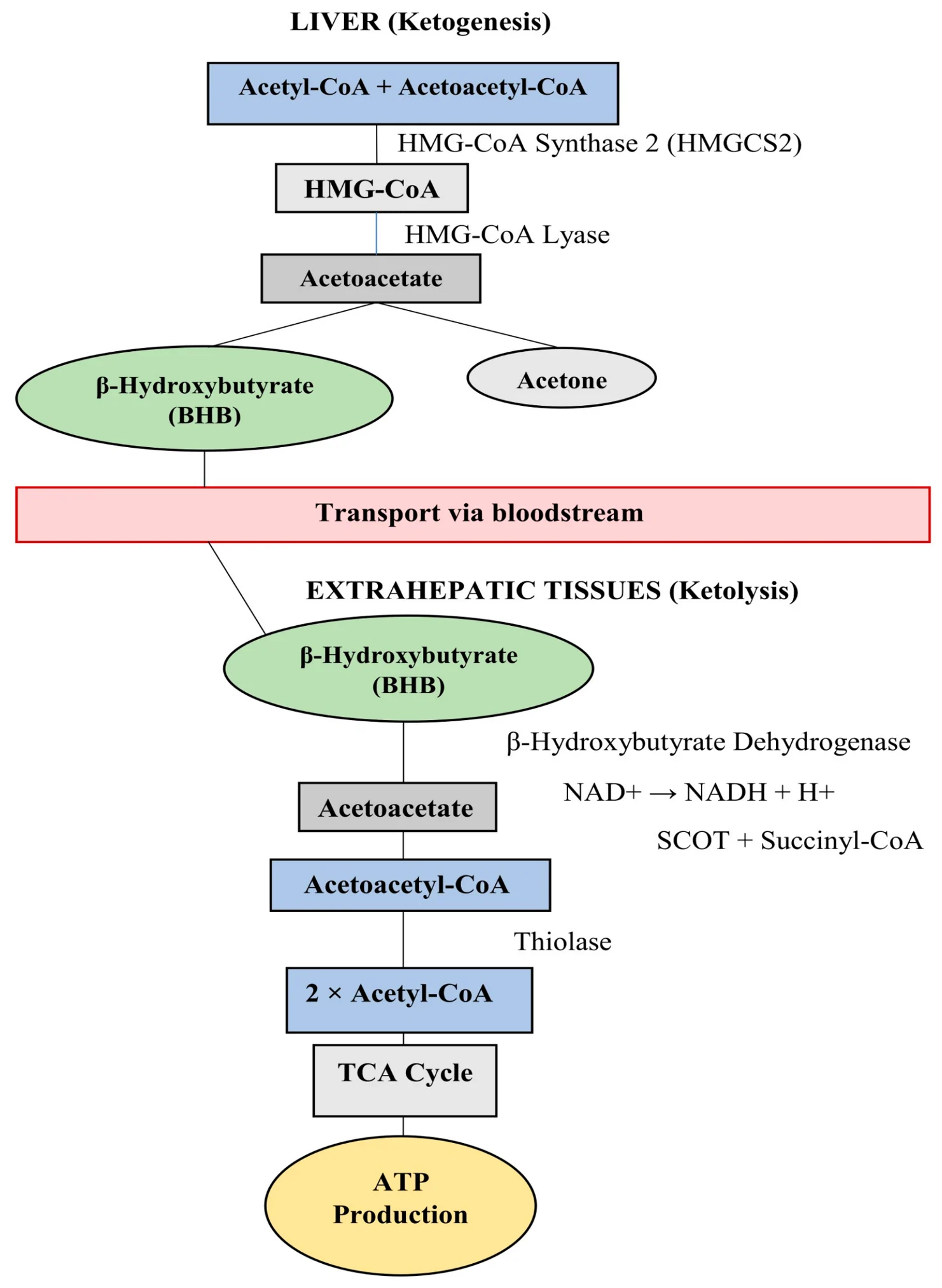
Ketogenesis and ketolysis pathway [9,10]. LIVER—key enzyme: HMG-CoA Synthase 2 (HMGCS2), generates acetoacetate, β-hydroxybutyrate (BHB), and acetone, lacks the enzyme SCOT, so it cannot utilize ketone bodies for energy; EXTRAHEPATIC TISSUES—key enzyme: SCOT (Succinyl-CoA:3-oxoacid CoA transferase); convert ketone bodies into acetyl-CoA, which enters the TCA cycle for ATP production; major ketone-consuming tissues include skeletal muscle, cardiac muscle, and the brain during fasting.

Figure 1 illustrates the pathway of ketogenesis occurring in the liver and the pathway of ketolysis occurring in other organs, such as skeletal muscle, the heart, and the brain.

The mitochondrial enzyme 3-hydroxy-3-methylglutaryl-CoA synthase (HMGCS2) plays a key role in the process of ketogenesis. It catalyzes the rate-limiting step of ketogenesis, namely the condensation of acetyl-CoA and acetoacetyl-CoA to form 3-hydroxy-3-methylglutaryl-coenzyme A (HMG-CoA) and free coenzyme A [11]. HMG-CoA is converted by HMG-CoA lyase into acetoacetate, which is reduced to β-hydroxybutyrate (BHB) or acetone [10]. Once ketogenesis is complete, the ketone bodies leave the liver, and ketolysis then occurs in target tissues—a process that cannot take place in the liver because the liver lacks succinyl-CoA:3-oxoacid CoA transferase (SCOT). In order for BHB to be used as an energy source, it must be oxidized to acetoacetate; during this process, oxidized nicotinamide adenine dinucleotide (NAD) is reduced to the reduced form of nicotinamide adenine dinucleotide (NADH) [9]. Next, SCOT catalyzes the activation of acetoacetate by adding coenzyme A from succinyl-CoA, resulting in the formation of acetoacetyl-CoA. Acetoacetyl-CoA is then cleaved by thiolase into two molecules of acetyl-CoA, which can enter the Krebs cycle in the target cell, enabling the synthesis of adenosine triphosphate (ATP) [9,10].

### 3.1. Variants of Ketogenic Diet

The MCT ketogenic diet is based on medium-chain fatty acids, which, compared to long-chain fatty acids (LCTs), are metabolized into ketone bodies much more quickly. In this dietary model, energy from MCT fats accounts for approximately 40–50% of total energy intake. Carbohydrates account for 15–18% of energy intake, while protein accounts for about 10%. Since most foods are dominated by LCTs and contain only small amounts of MCTs, patients often supplement their diet with MCT oil or emulsion-based preparations to achieve the required energy contribution from these fats [3].

The Atkins Modified Ketogenic Diet is considered a less restrictive alternative to the CKD. This diet allows for a higher protein intake compared to the CKD. This model does not impose strict restrictions on the amount or type of calories and protein consumed. The only component subject to restrictions is carbohydrates [3].

In MAD, carbohydrates are limited to approximately 20 g per day during the first month; thereafter, the intake may be increased by 5 g each month, but carbohydrates should not exceed 30 g per day in adults and 20 g per day in children. MAD is characterized by better patient acceptance than CKD. Mutarelli et al. [12] state that the use of MAD in combination with pharmacotherapy is associated with a significant reduction in epileptic seizures in both adults and children. The low-glycemic-index ketogenic diet (LGIT), similar to MAD, is a less restrictive and more accessible variation of the ketogenic diet.

The LGIT is primarily based on foods with a low glycemic index of less than 50 [13]. This dietary model allows for a daily carbohydrate intake of 40 to 60 g. Protein accounts for about 30% of total energy intake, while fat, as the primary energy source, accounts for 60% of daily caloric needs. The LGIT is not designed to induce a constant, deep state of ketosis, but rather to maintain stable blood glucose levels by preventing sharp spikes in insulin [3].

**Figure 2:**
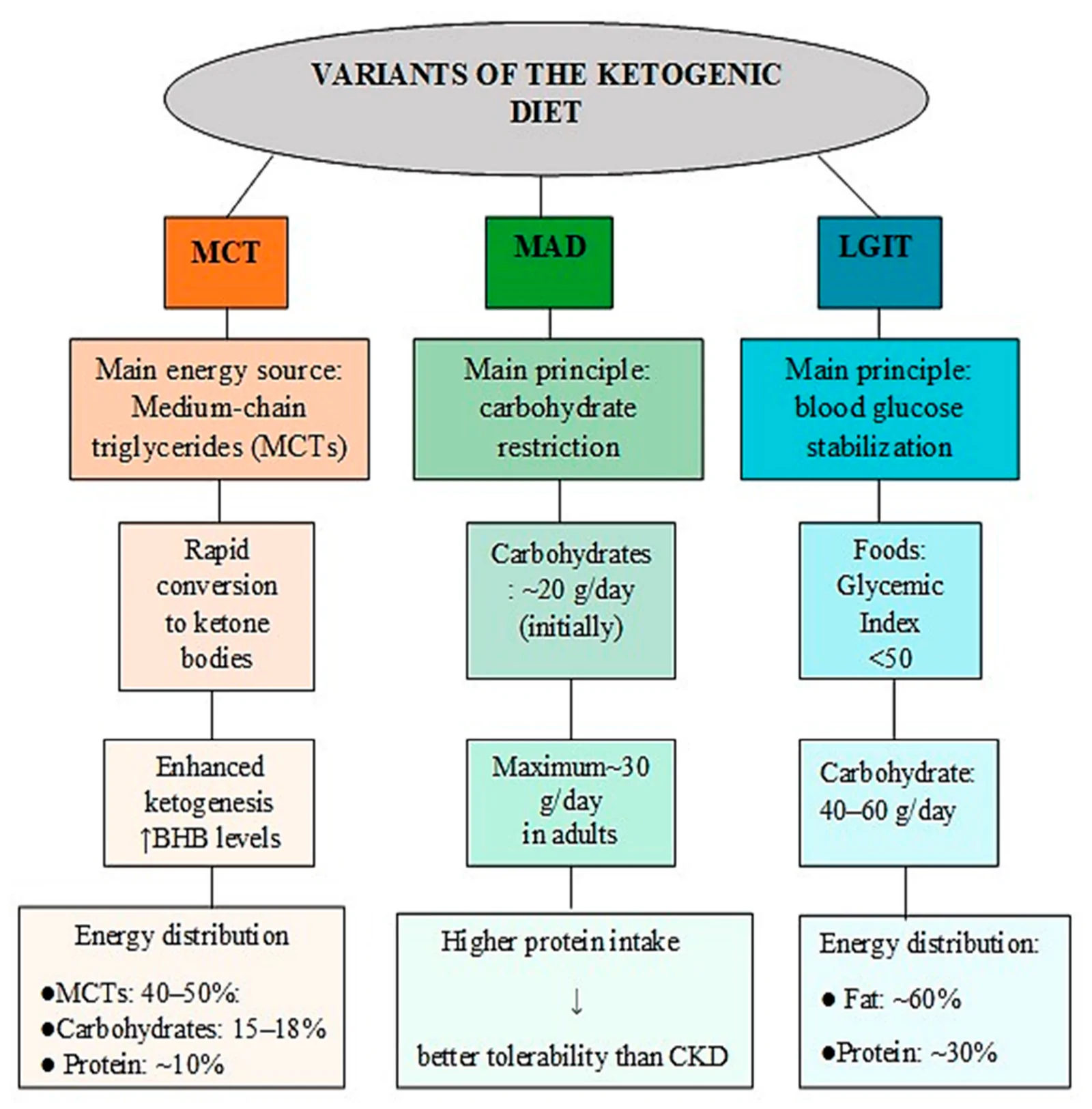
Characteristics of selected variants of ketogenic diets (MCT, MAD and LGIT) [3,12]. MCT Diet—Medium-Chain Triglyceride Diet; MAD—Modified Atkins Diet; LGIT—Low Glycemic Index Treatment; CKD—Classical Ketogenic Diet; BHB—β-Hydroxybutyrate.

Figure 2 shows the most popular variants of the ketogenic diet.

### 3.2. Potential Applications of the Ketogenic Diet

The use of the ketogenic diet and its variations is most well-documented in cases of drug-resistant epilepsy. Current research suggests that ketogenic diet therapy, along with its variations, also shows potential benefits in neurological and metabolic disorders, as well as in oncology [3]. The classic ketogenic diet and its variations have documented clinical applications in drug-resistant epilepsy in children [3]. In children and adolescents following the ketogenic diet or its variations in combination with pharmacotherapy, a reduction in epileptic seizures has been observed, and it has been suggested that the diet, when combined with pharmacological treatment, may provide therapeutic benefits [14]. Lakshminarayanan et al. [15] conducted a randomized trial comparing antiepileptic drug therapy to antiepileptic drug therapy supplemented with the LGIT diet in children aged 2–8 years. The study lasted 3 months and included 40 children. They were divided into two groups of 20 each, with the treatment group following the LGIT diet and the control group receiving no dietary recommendations. The study demonstrated that the diet used in the treatment group yielded better clinical outcomes. Of the 20 patients, six showed a > 50% reduction in epileptic seizures, one patient showed a >90% reduction in epileptic seizures, and another showed a complete cessation of epileptic seizures within 3 months of the study’s start.

Currently, there is no effective medication for treating glucose transporter 1 deficiency syndrome (GLUT1 DS), but CKD and MAD may help preserve brain growth and function and should therefore be implemented as soon as possible after a diagnosis of GLUT1 DS [16]. GLUT1 DS is a rare genetic disorder characterized by impaired energy metabolism in the brain [17]. The ketogenic diet is also used in the treatment of pyruvate dehydrogenase complex deficiency (PDHC). In some medical facilities, the ketogenic diet is recommended as part of a treatment regimen that, when combined with antiepileptic drugs, may provide therapeutic benefits [18,19]. A case report by Inui et al. [20] confirms that an intravenously administered ketogenic diet may be a therapeutic option for newborns with this deficiency.

Alzheimer’s disease is a neurodegenerative brain disorder that destroys nerve cells through the pathological accumulation of proteins inside brain cells and in the extracellular space, resulting in low levels of neurotransmitters [21]. The ketogenic diet and its variations have the potential to improve neural plasticity and cognitive function in people with Alzheimer’s disease [22]. Studies have shown that ketone bodies can improve short-term, episodic memory in people in the early stages of the disease and enhance quality of life [23]. The ketogenic diet also represents a promising therapeutic strategy for Parkinson’s disease. According to a study conducted by Anwar et al. [24], patients following a ketogenic diet showed improvements in motor and cognitive symptoms.

The ketogenic diet may be used as an adjunct therapy to standard pharmacotherapy for type 2 diabetes, overweight, or obesity. As noted by Zhou et al. [25], a meta-analysis found that groups following a ketogenic diet experienced a significant reduction in body weight and waist circumference compared to groups not following a ketogenic diet. The authors also demonstrated significant decreases in glycated hemoglobin (HbA1c) and triglycerides, as well as improved cellular insulin sensitivity.

The ketogenic diet may potentially be used as an adjunctive therapy for cancer patients. In a meta-analysis by Yang et al. [26] noted that patients reported higher levels of satisfaction with their treatment compared to the control group. A study conducted by Khodabakhshi et al. [27] showed that female breast cancer patients on a ketogenic diet reported a higher quality of life.

In summary, nutritional intervention using variations of the ketogenic diet has been most extensively studied in drug-resistant pediatric epilepsy and is an important component of therapy for rare metabolic disorders, such as GLUT1 DS and PDHC deficiency syndrome. Preclinical and clinical studies show that the ketogenic diet may find broader application in neurology, type 2 diabetes, and oncology.

## 4. Cancer and Ketogenic Diet

### 4.1. Molecular Basics of Oncogenesis

According to the National Cancer Institute, cancer is a disease in which mutated cells divide uncontrollably and can metastasize to other tissues [28,29]. For metastasis to occur, molecular changes must take place. Mutations in a cell lead to the formation of oncogenes that is, mutated proto-oncogenes responsible for regulating cell division. Proto-oncogenes encode proteins that stimulate proliferation, whereas oncogenes are mutated proto-oncogenes that cause the proteins responsible for cell division to malfunction, resulting in disrupted cell division. The loss of a cancer cell’s responsiveness to regulatory factors leads to the formation of descendant cell lines characterized by completely disrupted division control. Uncontrolled cell division requires the inactivation of the retinoblastoma protein (pRb). The pRb protein regulates the cell’s transition from the G1 phase to the S phase, i.e., the synthesis phase. In its active state, the pRb protein inhibits the E2F transcription factors, which are essential for entry into the S phase. The transforming growth factor-beta (TGF-β) suppressor factor, by binding to its receptors, induces the expression of the inhibitors p15INK4B and p21 [30].

These proteins inhibit the activity of the cyclin D and CDK4 kinase enzyme complexes, thereby preventing the inactivation of pRb. The loss or mutation of TGF-β in cancer cells disrupts this signaling pathway. The lack of inhibition allows the cyclin D and CDK4 kinase complexes to inactivate the pRb protein. This results in the release of E2F factors, leading to uncontrolled progression of the cell into the S phase and rendering the cell insensitive to suppressor signals. In turn, cancer cells evade differentiation through the overexpression of the c-Myc oncogene. This process shifts the intracellular balance in favor of uncontrolled growth, effectively blocking differentiation signals. This results in an increased energy demand in cancer cells. Furthermore, cancer cells become resistant to apoptosis, for example, through the loss of function of the p53 protein [31].

This allows the cell to undergo further mutations without the risk of self-destruction. The Hayflick limit is a term used to describe the maximum number of cell divisions; for a cancer cell to achieve uncontrolled and unlimited division, it must bypass the Hayflick limit. This occurs through the activation of the enzyme telomerase (in most cases) or through a mechanism that lengthens telomeres, thereby maintaining them above a critical length. This enables cancer cells to divide uncontrollably [31].

A cell’s ability to divide effectively requires constant external stimulation (e.g., via signal transduction). Growth factors that bind to specific receptors—such as tyrosine kinase receptors—on the cell membrane play a key role. The next stage involves receptor activation and signal transmission via signaling protein cascades. Of particular importance are the Ras/MAPK and PI3K/Akt pathways, as well as the Ras protein. The signal ultimately reaches the cell nucleus, where it modulates the expression of genes responsible for the cell cycle. Cancer cells are characterized by the deregulation of this process [32].

Growth factors play a particularly important role in signal transduction. From the perspective of dietary therapy, insulin-like growth factor 1 (IGF-1) and insulin are especially important growth factors. Blood insulin levels are closely linked to carbohydrate intake. When IGF-1 binds to its specific receptor—IGF-1R—it is activated, which initiates the PI3K/AKT/mTOR and Ras/Raf/MEK pathways. These two pathways are responsible for promoting cell proliferation and inhibiting apoptosis [33].

The mTOR (mechanistic Target of Rapamycin) protein is responsible in cellular physiology for receiving and integrating signals from the external environment, which allows for the regulation of metabolism. Its function is linked to the PI3K/Akt pathway. When a cell receives a signal from growth factors via receptors on its cell membrane, it activates the PI3K kinase, which then transmits the signal to mTOR through other proteins. Ribosomes are stimulated by mTOR to synthesize proteins. In cancer cells, a loss of control over mTOR is observed. This protein becomes overactive, leading to uncontrolled growth even under unfavorable environmental conditions surrounding the cell [32].

### 4.2. Basics of Warburg Effect

Under aerobic conditions, during glycolysis, glucose is broken down into pyruvate, which is transported to the mitochondria, where it enters the Krebs cycle. In cancer cells, however, even when oxygen is available, glucose is converted into lactate (this is known as the Warburg effect). The difference in the efficiency of this process is enormous, as cancer cells generate only 2 ATP molecules per glucose molecule, in contrast to non-mutated cells, which generate as many as 36 ATP molecules per glucose molecule. Due to their low energy efficiency, cancer cells are forced to increase their uptake of glucose from the extracellular environment. Cancer cells preferentially utilize glycolysis because they use its intermediates as precursors for the synthesis of lipids and nucleotides. The classic Warburg hypothesis posited that cancer cells have damaged mitochondria and are unable to utilize energy from ketone bodies and fatty acids unlike cells with intact mitochondria, which have the ability to shift their substrate preferences from glucose to other energy sources. Contrary to Warburg’s classic assumptions about irreversible mitochondrial dysfunction, the current scientific literature shows that mitochondria in cancer cells are functional and metabolically adapted to the tumor environment [32].

For cancer cells, glycolysis, in addition to generating ATP, also enables increased proliferation. By inhibiting the final stage of glycolysis, it allows the cancer cell to accumulate glycolytic intermediates—that is, intermediate products of glycolysis—which are precursors to macromolecules, including lipids and nucleotides. Glycolytic intermediates play a key role in the production of biomass, which is essential for tumor progression [32]. Cancer cells require a substantial and constant supply of glucose, so it seems reasonable to strictly limit carbohydrate intake. Therefore, a ketogenic diet, which is characterized by limiting carbohydrate intake to a minimum, may offer potential benefits. Reducing carbohydrate intake to a minimum causes a drop in blood glucose levels, which theoretically creates an unfavorable environment for the growth of cancer cells. A significant decrease in glucose results in a decrease in insulin and IGF-1 levels—growth factors responsible for promoting tumor growth [9].

**Figure 3:**
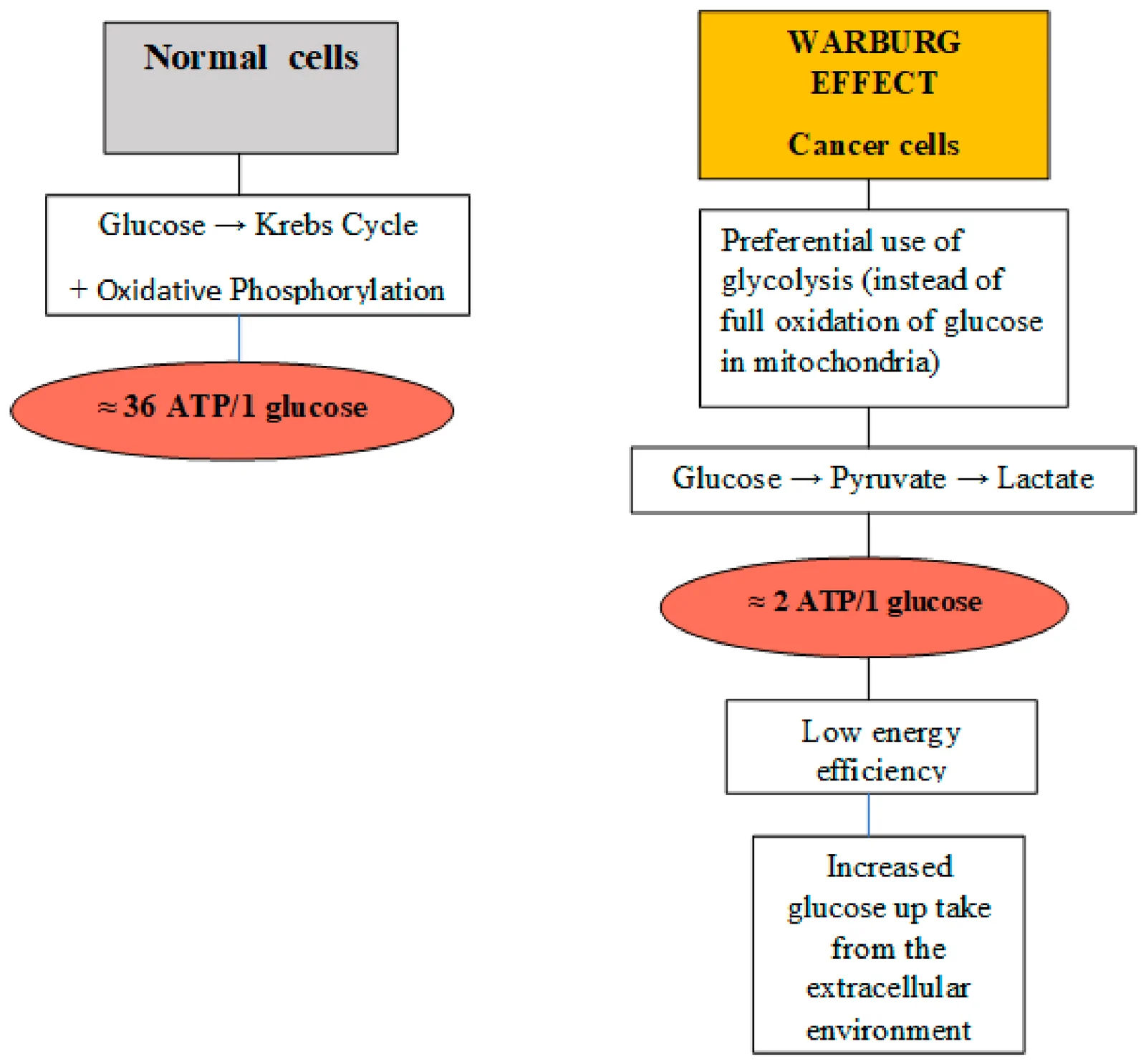
Glycolysis in cancer cells (the Warburg effect) and healthy cells [32].

Figure 3 compares the Warburg effect in cancer cells with glucose utilization by normal cells.

In a meta-analysis by Zhang et al. [34], it was noted that glucose transporter type 1 (GLUT1) was overexpressed on the cell membranes of lung adenocarcinoma (LUAD) cells. To compensate for inefficient metabolism, cancer cells increase the number of GLUT1 molecules, which form a channel in the cell membrane, allowing for increased glucose uptake into the cell.

GLUT1 overexpression is widely utilized in oncological Positron Emission Tomography (PET) diagnostics, which relies on the intense uptake of glucose analogs by cancer cells [32].

In an animal model, it was demonstrated that ketone bodies, specifically BHB, act as inhibitors of histone deacetylase (HDAC). HDAC enzymes are responsible for silencing the transcription of FOXO3A and the promoter of the metallothionein 2 gene (Mt2), which under physiological conditions constitute a cellular defense mechanism against reactive oxygen species (ROS). Cancer cells often exhibit overactivity of HDAC inhibitors, which suppress the activity of tumor suppressor genes. As an inhibitor, BHB blocks the activity of HDAC, thereby unblocking the tumor suppressor genes. Tumor suppressor genes inhibit cell proliferation and can lead to apoptosis. Tumor suppressor genes neutralize the effects of ROS, which are essential for the functioning of cancer cells [35].

A meta-analysis by Wang et al. [36] shows that higher levels of FOXO3A expression are associated with prolonged overall survival, a lower risk of lymph node metastasis, a lower tumor stage, and a lower likelihood of distant metastases in patients with the solid tumors discussed in the meta-analysis (including gastric, breast, and ovarian cancer).

### 4.3. Metabolic Reprogramming by Cancer Cells

A common misconception among cancer patients is the belief that cancer cells derive their energy solely from glucose and that a ketogenic diet can “starve” them. Cancer cells are highly adaptable. As described by Pavlova et al. [37], cancer cells are capable of utilizing alternative energy sources under extremely adverse conditions. These sources include fatty acids, proteins, glutamine, and acetate. In the case of certain cancers, acetate is taken up from the plasma and converted by acyl-CoA synthetase (ACSS2) into acetyl-CoA, which plays a key role in the Krebs cycle.

The reprogramming of energy metabolism is one of the key characteristics of cancer cells. Uncontrolled cell division results in increased cellular energy demand. By adapting their energy metabolism, cancer cells maintain uninterrupted tumor progression. They are capable of glycolysis even in the presence of oxygen and can utilize glycolytic intermediates for their metabolic pathways [38].

It is worth noting that mitochondria are extremely important for cancer cells. Otto Warburg proposed the theory that most cancer cells have damaged mitochondria. Current scientific literature suggests that most cancer cells have functioning mitochondria. An animal model has shown that cancer cells in which mitochondrial DNA (mtDNA) was intentionally damaged exhibited significantly delayed tumor growth; however, the damaged mtDNA was replaced by mtDNA from the surrounding healthy host cells [39].

### 4.4. Potential Application of Ketogenic Diet in Oncology

The potential application of the ketogenic diet in oncology has been investigated both in clinical studies involving cancer patients and in preclinical studies using animal models. The available evidence suggests that the ketogenic diet may influence body composition and selected metabolic parameters in cancer patients; however, its clinical efficacy remains inconclusive and may depend on the individual characteristics of both the patient and the tumor.

#### 4.4.1. Evidence from Studies in Humans

Zhao et al. [40] observed weight loss in patients with breast, ovarian, and pancreatic cancer following a ketogenic diet. The reduction in body weight was primarily attributable to a decrease in body fat rather than muscle mass. Importantly, the intervention was not associated with deterioration in kidney function, as no statistically significant changes in creatinine or blood urea nitrogen levels were observed. Similarly, no significant changes in alanine aminotransferase (ALT) or albumin levels were reported, and no deterioration in parameters indicative of liver or kidney dysfunction was identified.

These findings are consistent with a review by Kamali et al. [41], which reported reductions in total body weight and body fat while preserving lean body mass in patients with solid tumors, including head and neck cancers and early-stage breast cancer. From a metabolic perspective, the review indicated that adherence to a ketogenic diet may be associated with reductions in blood glucose, triglyceride, and insulin-like growth factor 1 (IGF-1) levels. However, the authors emphasized that metabolic responses among cancer patients are highly variable.

Despite these potentially beneficial effects, the clinical evidence remains insufficient to establish the ketogenic diet as an effective component of cancer treatment. Römer et al. [42] emphasized that there is currently no conclusive evidence confirming a positive therapeutic effect of the ketogenic diet in oncology. Therefore, the diet should not be introduced routinely without appropriate medical supervision and individualization according to the patient′s clinical condition, nutritional status, and type of cancer.

Another potential application of the ketogenic diet is to support patients during conventional cancer treatment. Patients undergoing chemotherapy or radiotherapy frequently experience treatment-related adverse effects and deterioration in quality of life (QoL) [43]. Some evidence suggests that the ketogenic diet may increase the tolerance of healthy cells to the cytotoxic effects of anticancer therapies and consequently potentially reduce treatment-related adverse effects [41]. However, this potential benefit requires further confirmation in well-designed clinical trials.

#### 4.4.2. Evidence from Animal Studies

A greater body of evidence concerning the potential anticancer mechanisms of the ketogenic diet has been obtained from preclinical studies. A meta-analysis of studies conducted in animal models, including rats and mice, demonstrated that the use of a ketogenic diet was associated with a statistically significant prolongation of survival and a reduction in tumor volume [44]. These findings suggest that the ketogenic diet may have potential as an adjunct to conventional anticancer treatment rather than as a standalone therapy.

Preclinical studies have also indicated that the ketogenic diet may enhance the response of tumor cells to chemotherapy and radiotherapy. Its combination with standard treatment was associated with increased sensitivity of cancer cells to conventional therapies. An enhanced response to targeted therapies, including phosphoinositide 3-kinase (PI3K) inhibitors, has also been observed in certain tumor models [44].

The ability of the ketogenic diet to inhibit tumor growth has additionally been demonstrated in animal models of neuroblastoma. In nude mice, inhibition of neuroblastoma progression was positively associated with reduced blood glucose levels. This effect was accompanied by decreased expression of Ki-67 and phospho-histone H3 (pHH3), which are established markers of cellular proliferation. These findings indicate that metabolic alterations induced by the ketogenic diet may influence tumor cell proliferation. Importantly, the response to the ketogenic diet appears to depend on the metabolic and genetic characteristics of the tumor. The susceptibility of tumor cells to metabolic interventions may be related, among other factors, to the degree of mitochondrial dysfunction. Tumors originating from neuroepithelial tissue of the central nervous system may exhibit increased dependence on glucose due to impaired respiratory-chain activity, potentially making them more susceptible to glucose-restricting interventions such as the ketogenic diet. Conversely, not all tumors respond favorably to the ketogenic diet. In an animal model, the highly aggressive SK-N-BE(2) neuroblastoma cell line, characterized by a mutation in the TP53 tumor suppressor gene and amplification of the NMYC oncogene, demonstrated resistance to ketogenic diet intervention. This resistance was associated with the ability of the tumor cells to utilize glutamine as their primary energy substrate. Researchers suggested that NMYC amplification may promote increased glutamine uptake and its utilization as an alternative energy source, thereby reducing the effectiveness of glucose restriction [45].

Overall, the available evidence indicates that the ketogenic diet may have potential as a supportive or adjunctive strategy in oncology. Human studies suggest that it can contribute to reductions in body weight and body fat while maintaining lean body mass, without clear evidence of deterioration in liver or kidney function [40,41]. However, clinical evidence of direct anticancer efficacy remains insufficient [42]. In contrast, preclinical studies provide more promising evidence of reduced tumor growth, prolonged survival, and increased sensitivity to conventional and targeted therapies [44,45].

Nevertheless, the heterogeneous response observed between different tumor types highlights the importance of considering the metabolic and genetic characteristics of individual tumors. Further well-designed clinical trials are therefore required before the ketogenic diet can be routinely recommended as part of cancer treatment.

Table 1 shows summary of the evidence on the potential application of the ketogenic diet in oncology.

## 5. Challenges Associated with the Use of the Ketogenic Diet as an Adjunctive Therapy

### 5.1. Methodological Limitations

Despite increasing interest in the ketogenic diet in oncology, its clinical application remains controversial, and the quality of the available evidence is insufficient to establish its efficacy. Several methodological and practical limitations complicate the interpretation of current findings.

One of the major concerns is the difficulty in determining whether the observed effects are attributable specifically to carbohydrate restriction and ketosis or rather to an overall reduction in energy intake. Szendi et al. [46] highlighted that some of the benefits attributed to the ketogenic diet may be related to caloric restriction rather than to ketosis itself. This represents an important methodological issue, particularly in studies in which total energy intake is not adequately controlled.

The quality of clinical evidence is another major limitation. Although the evidence from epilepsy research cannot be directly extrapolated to oncology, the systematic review by Mc Gill et al. [5] illustrates broader methodological problems associated with ketogenic diet studies, including small sample sizes and short intervention periods. Similar limitations have been reported in oncology research. Schmidt et al. [47] noted insufficient evidence to confirm the clinical efficacy of ketogenic diet in cancer patients, with concerns regarding inadequate randomization, limited sample sizes, and low methodological reliability. Furthermore, intensive nutritional counseling and clinical support provided to participants may act as confounding factors, making it difficult to determine whether observed benefits result specifically from the ketogenic macronutrient composition.

Another important limitation is poor adherence to the diet. High dropout rates have been reported, partly because of difficulties maintaining a highly restrictive dietary pattern and because of treatment-related or diet-related adverse effects. Reported adverse events include nausea, fatigue, gastrointestinal disturbances, and, in some circumstances, hypoglycemia [47]. These factors may introduce attrition bias and further limit the generalizability of study findings.

The lack of standardized ketogenic protocols represents an additional challenge. As noted by Reichel et al. [48], some oncology trials have adopted ketogenic diet protocols originally developed for epilepsy or obesity rather than protocols specifically designed according to tumor type, treatment modality, nutritional status, and metabolic characteristics of cancer patients. Differences in macronutrient composition, caloric intake, duration of intervention, and target ketone levels make comparisons between studies difficult. Finally, frequent monitoring of blood glucose and ketone levels may impose an additional burden on patients, particularly those undergoing intensive anticancer treatment. This may negatively affect adherence and feasibility in routine clinical practice.

Overall, these methodological and practical limitations substantially restrict the interpretation of the current evidence. Future studies should employ adequately powered randomized controlled designs, standardized and cancer-specific ketogenic diet protocols, appropriate control groups, clearly defined adherence criteria, and clinically meaningful endpoints.

Table 2 shows main methodological and clinical limitations of ketogenic diet studies in oncology.

### 5.2. Side Effects and Metabolic Risks of the Ketogenic Diet

The ketogenic diet is characterized by a high proportion of dietary fat, which may account for up to 90% of total energy intake depending on the specific protocol [3]. Such a dietary pattern may induce clinically relevant metabolic changes, particularly in lipid metabolism, and may also be associated with gastrointestinal, nutritional, and metabolic adverse effects.

One of the main concerns is the potential impact of ketogenic diet on the lipid profile. A meta-analysis by Choi et al. [49] demonstrated that ketogenic diet was associated with increased total cholesterol (TC) and low-density lipoprotein cholesterol (LDL-C) concentrations in overweight or obese individuals without type 2 diabetes. The authors also reported changes in LDL particle characteristics, including a reduction in the proportion of small, dense LDL particles. However, these findings should be interpreted in the context of the overall changes in lipid metabolism induced by ketogenic diet.

Similarly, Burén et al. [50], in a tightly controlled dietary intervention involving 17 healthy young women, observed significant increases in LDL-C and apolipoprotein B-100 (ApoB-100) following a ketogenic low-carbohydrate, high-fat diet. In contrast to the findings of Choi et al. [49], the study reported an increase in small, dense, potentially atherogenic LDL particles. Although the sample size was small, the controlled provision of meals strengthens the methodological control over dietary exposure.

These findings may be particularly relevant in breast cancer patients because some anticancer therapies can independently affect cardiovascular risk. Aromatase inhibitors may adversely influence lipid parameters, whereas anthracyclines are associated with well-established cardiotoxicity [51,52]. Therefore, the potential cardiovascular consequences of ketogenic diet should be considered when it is introduced in patients receiving therapies with known cardiovascular effects. However, evidence demonstrating a direct synergistic interaction between ketogenic diet and these treatments remains insufficient, and this potential risk requires further investigation.

In addition to metabolic effects, ketogenic diet is associated with a range of adverse events. A systematic review by Schopf et al. [53] reported that 43% of participants experienced at least one adverse effect. Gastrointestinal symptoms, including constipation, vomiting, diarrhea, and nausea, were among the most frequently reported complications. Other reported effects included taste disturbances, hyperuricemia, metabolic acidosis, dehydration, hypoglycemia, electrolyte disturbances, and neurological symptoms. The frequency and severity of these effects may vary according to the type of ketogenic protocol, patient population, and duration of dietary intervention.

Adherence represents another important limitation. The restrictive nature of ketogenic diet, together with appetite loss, anorexia, and gastrointestinal symptoms, may contribute to treatment discontinuation. Differences in adverse-event profiles between age groups have also been reported. For example, infections represented a greater proportion of reported adverse events in pediatric than in adult populations; however, a direct causal relationship with ketogenic diet has not been established [53].

Nutritional inadequacy represents an additional concern, particularly when ketogenic diet is implemented without appropriate dietary planning. Restriction of whole grains, fruits, and some vegetables may reduce the intake of dietary fiber and several micronutrients. Studies of high-fat low-carbohydrate and ketogenic diets have reported potential deficiencies in folate, vitamins B1, B6, B12, A, E, and K, as well as calcium, magnesium, potassium, iron, and dietary fiber [7,54]. Importantly, the nutritional adequacy of ketogenic diet depends on the specific formulation of the diet; contemporary protocols may incorporate greater amounts of low-carbohydrate vegetables than older classic or modified ketogenic models. Therefore, deficiencies should not be assumed to occur uniformly across all ketogenic diet protocols.

These concerns are particularly important in patients with cancer, who may already be at risk of malnutrition, weight loss, sarcopenia, electrolyte disturbances, and micronutrient deficiencies as a consequence of the disease and its treatment. Consequently, ketogenic diet should not be implemented without appropriate nutritional assessment and medical supervision. In oncology settings, individualized dietary planning and monitoring of nutritional status, hydration, electrolytes, glucose, and ketone levels may be necessary to minimize potential risks.

Table 3 shows side effects and metabolic risks associated with the ketogenic diet.

## 6. Oncological Aspects of the Ketogenic Diet in Female Breast Cancer Patients

### 6.1. Breast Cancer and Its Biological Subtypes

Breast cancer is the most common malignancy affecting women over 45 years of age. The incidence of breast cancer in children and men is extremely rare [55]. Breast cancer is characterized by metastasis most commonly to the bones, lungs, liver, and brain [56]. This metastases are the primary cause of treatment failure and mortality in individuals with breast cancer [57]. Breast cancer is not a single disease entity; it is divided into multiple types and biological subtypes. Defining the specific biological subtype of breast cancer is crucial, as it may differ in its clinical course, phenotype, and metabolic genotype [58]. Four main biological subtypes of breast cancer are distinguished: the luminal A subtype, the luminal B subtype, the HER2-positive (human epidermal growth factor receptor 2) subtype, and the most aggressive triple-negative subtype [56].

The luminal A subtype belongs to a group of tumors with numerous estrogen receptors [56]. It is the most common breast cancer subtype. It is characterized by a low Ki-67 index and expresses the progesterone receptor [55]. The luminal B subtype is characterized by a higher Ki-67 proliferation index and lower expression of hormone receptors compared to the luminal A subtype. Additionally, the luminal B subtype has a poorer prognosis than the luminal A subtype [56]. Unlike the luminal A subtype, it lacks the progesterone receptor [55].

The HER2-positive subtype is characterized by HER2 overexpression [56]. According to the BCRF (Breast Cancer Research Foundation), overexpression of the HER2 protein occurs in 25–30% of breast cancers. From a clinical perspective, HER2 overexpression is a feature associated with poorer treatment outcomes. This subtype is considered aggressive [59]. However, it is worth noting that anti-HER2 therapies improve the prognosis for patients [56].

The triple-negative breast cancer subtype (TNBC) is considered the most aggressive. It is characterized by a lack of expression of progesterone and estrogen receptors, as well as an absence or low expression of the HER2 protein [55]. It is associated with hereditary mutations in the BRCA1 tumor suppressor gene, whose role involves maintaining genomic stability and DNA repair, and which is responsible for regulating the cell cycle [60]. It features an aggressive phenotype, which translates into a poorer prognosis and limits therapeutic options [61].

### 6.2. Metabolic Differences Between Breast Cancer Subtypes

Significant differences in the energy metabolism of biological breast cancer subtypes demonstrate how variably they can respond to specific nutritional interventions. A lack of personalized nutritional therapy may lead to outcomes contrary to those intended.

Differences in Ki-67 values between luminal A and B tumors may determine the response to a ketogenic diet intervention. The luminal A subtype is characterized by a low Ki-67 index, indicating that this breast cancer subtype has a slow proliferation rate [55]. A slower division rate may imply lower energy demands. Lower energy demands compared to the luminal B subtype may make it more resistant to glucose deprivation resulting from carbohydrate restriction during a ketogenic diet intervention.

The HER2-positive subtype is often characterized by increased activity of the fatty acid synthase (FASN) enzyme. While the acquisition of fatty acids for cell membrane construction is a universal cellular trait, individual breast cancer subtypes differ in their methods of obtaining them. Koundouros et al. [62] suggest that the HER2-positive subtype is strongly associated with de novo lipogenesis. Overexpression of the HER2 oncogene induces the constitutive activation of FASN. This represents a significant difference compared to, among others, TNBC, which prefers the uptake of exogenous lipids and is characterized by reduced FASN expression.

The application of a classic ketogenic diet (CKD), which assumes providing 90% of energy from fats, may yield a counterproductive effect in this context [63]. Such a drastic increase in fat supply could potentially lead to tumor cell progression rather than its inhibition.

The lack of progesterone, estrogen, and HER2 receptors in the TNBC subtype leaves patients with limited therapeutic options. Although a dominance of glycolysis is observed, nutritional intervention in the form of a ketogenic diet remains highly questionable. These doubts stem from the fact that the TNBC tumor subtype often exhibits overexpression of the MYC oncogene, which stimulates genes responsible for fatty acid oxidation (FAO). This helps the cell survive metabolic stress (e.g., glucose deprivation) through the generation of significant amounts of ATP [62,64].

Table 4 shows metabolic alterations in Luminal A, Luminal B, HER2-Positive and Triple-Negative Breast Cancer.

The aforementioned biological subtypes of breast cancer and the ability of tumor cells to reprogram their energy metabolism demonstrate that there is no universal therapeutic approach capable of guaranteeing identical clinical outcomes regardless of the biological subtype of the tumor. Metabolic diversity dictates that nutritional interventions in oncology patients must be treated with the same level of importance as pharmacological therapies. The specific biological subtype of breast cancer determines whether, and which, pharmacological therapy and nutritional intervention have a chance of success. The ketogenic diet is a potential supportive nutrition strategy for selected breast cancer patients. Its beneficial effects stem from, among other things, limiting glucose intake and modulating the metabolism of insulin, IGF-1, and ketone bodies. Available clinical trials have used varying macronutrient ratios. Breast cancer molecular subtype can be considered as an element of personalized intervention, but there are currently no clinical trials establishing distinct macronutrient ratios for ER+/PR+, HER2+, and TNBC. In the case of ER+/PR+ tumors, the metabolic phenotype, including obesity and insulin resistance, may be particularly important. In HER2+ and TNBC, the use of the Ketogenic Diet remains experimental, and interest in TNBC stems primarily from observed metabolic differences and preclinical data. Regardless of the tumor subtype, assessment of nutritional status, muscle mass, energy requirements, and treatment tolerance should be the basis for selection. In patients with malnutrition, sarcopenia, cachexia, or unintentional weight loss, ensuring adequate energy and protein intake is a priority, and a restrictive ketogenic diet should not be used solely for the purpose of achieving ketosis [4]. Current data support personalizing the ketogenic diet primarily according to the patient′s nutritional status and metabolic phenotype, taking into account the molecular tumor subtype, rather than using rigid, subtype-specific macronutrient ratios. Prospective studies comparing standardized ketogenic diet protocols across different breast cancer subtypes are necessary [65].

### 6.3. The Impact of the Ketogenic Diet on Conventional Cancer Therapies

Female breast cancer patients often experience weight gain following chemotherapy [64]. The phenomenon of post-chemotherapy weight gain is not only dangerous due to the reduction in patients′ quality of life (QoL), but it may also increase the risk of recurrence and mortality [66]. The primary goal of conventional oncological treatments, such as radiotherapy and chemotherapy, is to damage the genetic material of tumor cells or disrupt their cell cycle. Radiotherapy and certain cytostatic drugs utilize oxidative stress and the production of reactive oxygen species (ROS) for this purpose, whereas most chemotherapy drugs act by interfering with proliferative enzymes and the nucleic acid structure of cancer cells [58]. Ketone bodies inhibit ROS production by activating the Nrf2 (nuclear factor erythroid 2-related factor 2) signaling pathway, which is involved in the synthesis of endogenous antioxidants. Furthermore, by inhibiting HDAC, ketone bodies directly contribute to promoting the transcription of detoxification genes [67]. Insulin resistance is considered one of the primary side effects of chemotherapy and anti-hormonal therapy [64]. It is worth noting that drugs such as mTOR inhibitors can increase cellular insulin resistance, and may additionally cause potential hyperglycemia and disrupt the lipid profile by increasing blood concentrations of total cholesterol (TC) and triglycerides (TG) [68,69].

The side effects of conventional breast cancer treatment demonstrate that the use of various nutritional interventions as adjuvant therapies is justified. The argument for the ketogenic diet as a nutritional intervention to alleviate the side effects of oncological treatment is supported by the literature. In a meta-analysis by Kamali et al. [41], a beneficial reduction in adipose tissue was observed while maintaining lean body mass. Fat reduction was noted in overweight patients with stage I breast cancer. On the other hand, the authors emphasize that weight loss is not always the desired outcome. For example, in advanced stages of breast and lung cancer, particular attention must be paid to patient body weight, as cachexia represents a significant risk factor, especially at such an advanced stage of the disease. The ketogenic diet may reduce blood levels of TG and IGF-1, but the authors point out that the long-term use of the ketogenic diet was associated with a high dropout rate among oncology patients.

In oncology patients, obesity often leads—via excess adipose tissue—to increased blood concentrations of pro-inflammatory cytokines and interleukins, which result in tumor progression and lead to cellular insulin insensitivity. A reduction in body fat was also observed by Zhang et al. in their meta-analysis. Additionally, it has been suggested that the ketogenic diet may be associated with potential health benefits. The authors indicate that the ketogenic diet can improve the lipid profile by reducing LDL and TC, affect the endocrine system by lowering insulin and thyrotropin levels, and improve blood glucose levels [43].

A randomized clinical trial by Khodabakhshi et al. [70] analyzed the metabolic parameters of breast cancer patients undergoing chemotherapy. The study group was prescribed a ketogenic diet supplemented with MCT oil, while the control group followed a standard diet. The authors observed a statistically significant decrease in the concentration of tumor necrosis factor-alpha (TNF-α) in the study group, whereas the decrease in the control group was minimal. Another significant finding was the increase in interleukin-10 (IL-10) concentration in the study group, whereas it remained unchanged in the control group. An increase in IL-10 concentration is an undesirable phenomenon, as it is an immunosuppressive cytokine that can inhibit the body′s anti-tumor response. Furthermore, a decrease in fasting blood glucose (FBG) and lactate levels was observed in the study group, which led to a reduction in glycolysis—a shift that, according to the authors, may enhance the efficacy of oncological treatment. It should be noted, however, that this study raised numerous concerns, including a small sample size (*n* = 10), a high dropout rate, a lack of blinding, and a short study duration (12 weeks).

It is worth adding that there are preclinical studies demonstrating the potential synergism of a ketogenic diet intervention with conventional breast cancer treatments. One area of research is the synergy between the ketogenic diet and PI3K inhibitors. PI3K inhibitors block the insulin signaling pathway, but as a side effect, they cause hyperglycemia, which results in a significant insulin spike. The authors hypothesized that excess insulin, acting through insulin receptors, leads to the reactivation of the PI3K/mTOR pathway, effectively abolishing the therapeutic function of PI3K inhibitors. The mechanism of synergy between PI3K inhibitors and the ketogenic diet is based on eliminating this feedback-induced drug resistance. The reduction of hyperglycemia and the limitation of blood insulin levels through the ketogenic diet resulted in the inhibition of the PI3K/mTOR pathway in tumor cells. This led to a significant decrease in the Ki-67 protein and a clinically significant reduction in tumor mass and volume compared to therapy with PI3K inhibitors alone [71]. It must be noted that a ketogenic diet nutritional intervention is much easier to implement and control in an animal model than in a breast cancer patient following chemotherapy. The body of a patient after chemotherapy is severely weakened, which constitutes a significant translational barrier, preventing the experimental model from being replicated in a clinical population.

### 6.4. Cardiovascular Safety and Micronutrients During the Ketogenic Diet

The use of a ketogenic diet in patients with breast cancer requires careful assessment of cardiovascular risk, as both the high-fat diet itself and anticancer treatment can affect the metabolic profile and cardiovascular system. This risk may be particularly significant in patients with obesity, insulin resistance, hypertension, dyslipidemia, or existing cardiovascular disease. In patients with hormone-dependent breast cancer taking aromatase inhibitors (AIs), attention should be paid to the possibility of worsening lipid profiles and increased cardiovascular risk. ESC guidelines indicate that AI therapy may be associated with an increased risk of dyslipidemia, hypertension, heart failure, and myocardial infarction. Therefore, lipid profiles and cardiovascular risk factors should be monitored during treatment [72]. Anthracyclines, on the other hand, are characterized by well-documented, dose-dependent, and cumulative cardiotoxicity. Therefore, risk assessment and cardiac monitoring in patients receiving these drugs are necessary in accordance with cardio-oncology recommendations [72,73].

However, it should be emphasized that there are currently no clinical studies quantifying the additional or synergistic cardiovascular risk resulting directly from combining CD with aromatase inhibitors or anthracyclines. Therefore, the percentage increase in risk resulting from CD alone cannot be determined. From a clinical perspective, CD should be considered a potential additional risk modifier, particularly in the case of significant increases in LDL-C or triglycerides. In patients with increased cardiovascular risk, a CD model based primarily on monounsaturated and polyunsaturated fats, such as olive oil, avocado, nuts, seeds, and fish, while limiting saturated fats, should be preferred. Before starting the CD, it is recommended to assess blood pressure, lipid profile, blood glucose/HbA1c, body weight, and cardiovascular history. Monitoring according to ESC recommendations should be used in patients receiving potentially cardiotoxic treatment, including—depending on risk—ECG, echocardiography, and cardiac biomarker testing [72,73].

Another significant issue is the risk of insufficient micronutrient intake. Limiting cereal products, legumes, some fruits, and other carbohydrate foods may reduce the intake of magnesium, potassium, calcium, folate, and some B vitamins, among others. This risk may be further increased in cancer patients with limited appetite, nausea, vomiting, diarrhea, or unintentional weight loss.

Therefore, before starting the CD, it is recommended to assess nutritional status and, depending on the clinical situation, measure electrolytes, magnesium, calcium, renal and liver function parameters, complete blood count, vitamin B12, folate, iron, and 25(OH)D. In patients whose diet does not provide an adequate supply of micronutrients, a multivitamin and mineral supplement providing approximately 100% of the reference daily intake may be considered. Additional supplementation should be individualized based on dietary intake, laboratory results, and clinical status, rather than routinely administered in pharmacological doses [74,75].

Particular attention should be paid to calcium and vitamin D in patients treated with aromatase inhibitors due to the increased risk of bone loss and fractures during hormone therapy. Fiber intake should be primarily provided by low-carbohydrate vegetables, avocados, nuts, and seeds. In cases of insufficient intake, soluble fiber supplementation may be considered, taking into account gastrointestinal tolerance. However, routine use of high doses of antioxidants during active anticancer treatment without a known deficiency or a specific clinical indication is not recommended [74,75].

Consequently, the ketogenic diet in breast cancer patients should be implemented as an intervention requiring individual risk assessment, not as a universal nutritional model. Particular caution is required in patients receiving anthracyclines, aromatase inhibitors, or other therapies with potential cardiovascular effects. At the same time, in patients with malnutrition, sarcopenia, or cachexia, the primary goal should be to ensure adequate energy and protein intake and prevent muscle loss. Therefore, the use of the ketogenic diet should take into account the molecular subtype of the tumor, the type of treatment, nutritional status, metabolic profile, and cardiovascular risk.

### 6.5. The Impact of the Ketogenic Diet on the Quality of Life in Breast Cancer Patients

Oncological treatment is frequently associated with adverse effects that may substantially impair daily functioning and quality of life (QoL), including pain, peripheral neuropathy, nausea and vomiting, fatigue, cachexia, and psychological distress [55]. In breast cancer patients, QoL is therefore an important patient-reported outcome (PRO), providing information that may complement conventional clinical measures of treatment tolerance and functional status [76,77].

However, QoL outcomes observed during dietary interventions should be interpreted cautiously because they may be influenced by several concurrent factors, including the type and intensity of anticancer treatment, baseline nutritional status, physical activity, gastrointestinal symptoms, psychological status, and changes in total energy intake. Several studies have investigated the potential effects of a ketogenic diet on QoL in breast cancer patients, with heterogeneous results. Importantly, the available evidence does not demonstrate a consistent or sustained improvement in QoL attributable specifically to carbohydrate restriction.

Khodabakhshi et al. [27] investigated the effects of an isocaloric ketogenic diet supplemented with medium-chain triglycerides (MCTs) in breast cancer patients receiving chemotherapy. The intervention provided approximately 6% of energy from carbohydrates, 19% from protein, and 75% from fat, with MCTs contributing approximately 20% of total energy intake. QoL was assessed using the EORTC QLQ-C30 and QLQ-BR23 questionnaires. After 6 weeks, the ketogenic diet group demonstrated statistically significant improvements in QoL and physical activity compared with the control group. However, these findings should be interpreted cautiously because the control group experienced a higher frequency of diarrhea during the same period, which may have independently impaired QoL scores. Thus, the observed between-group difference cannot be unequivocally attributed to the ketogenic intervention.

Furthermore, the longer-term results provide less support for a sustained QoL benefit. After 12 weeks, no statistically significant differences in QoL were observed between the ketogenic diet and control groups. An additional important confounding factor was the reduction in energy intake in the ketogenic diet group, which decreased from approximately 1743 to 1245 kcal/day. This reduction was attributed, at least in part, to decreased appetite associated with the MCT-supplemented ketogenic diet [27].

Consequently, it cannot be determined whether the short-term changes in QoL were related to carbohydrate restriction, ketosis, changes in dietary composition, or reduced energy intake. Energy restriction itself may affect body weight, fatigue, physical functioning and psychological well-being and should therefore be considered when interpreting QoL outcomes.

A similar issue applies to the study by Klement et al. [78], which evaluated ketogenic diet during radiotherapy in patients with early-stage breast cancer. The intervention consisted of a diet providing approximately 75–80% of energy from fat, with carbohydrate intake limited to a maximum of 50 g/day, supplemented with MCTs and a Master Amino Acid Pattern (MAP) formulation. The study included patients with different breast cancer subtypes. A modest improvement in QoL was observed during the final week of radiotherapy in the ketogenic diet group; however, this difference did not reach statistical significance. Therefore, the study does not provide evidence of a statistically confirmed QoL benefit of ketogenic diet. Importantly, the study suggests that ketogenic diet was feasible during radiotherapy and did not appear to exacerbate psychosomatic symptoms in patients with early-stage breast cancer. This finding may be clinically relevant, but should not be interpreted as evidence of therapeutic efficacy.

Kämmerer et al. [79] evaluated nutritional interventions during oncological rehabilitation in a non-randomized clinical study. Participants selected their dietary group themselves, including ketogenic diet, low-carbohydrate and standard diets. Of 152 women enrolled, 121 were included in the final analysis, with only 20 participants following the ketogenic diet intervention. Although a significant improvement in global QoL was observed in the ketogenic diet group, the interpretation of this finding is limited by the absence of randomization, self-selection of the dietary intervention, the small sample size, lack of control over physical activity, and previous experience with low-carbohydrate diets among some participants. These factors introduce substantial potential for selection and performance bias. Patients who voluntarily choose a ketogenic diet may differ from control participants in motivation, health-related behavior, dietary literacy, physical activity, expectations regarding the intervention, and psychological characteristics. Consequently, the observed improvement cannot be confidently attributed to the ketogenic diet itself.

The systematic review and meta-analysis by Zhang et al. [43], which included patients with different types of cancer, reported improvements in selected QoL-related outcomes, particularly fatigue and emotional functioning, as well as possible improvements in insomnia and social functioning. However, the authors emphasized important limitations, including the small number and low quality of available studies, lack of long-term follow-up, heterogeneity between interventions, and the inclusion of different cancer types within the same analyses. Therefore, these findings provide supportive but insufficient evidence for a specific QoL benefit of ketogenic diet in breast cancer.

#### 6.5.1. Interpretation of Short-Term and Long-Term Outcomes

Taken together, the available evidence suggests that potential improvements in QoL associated with ketogenic diet may be transient and highly dependent on study context. The most favorable results were observed over relatively short periods, whereas the randomized study by Khodabakhshi et al. [27] did not demonstrate a significant QoL advantage after 12 weeks. Similarly, the study by Klement et al. [78] demonstrated only a non-significant improvement at the end of radiotherapy.

This distinction between short- and long-term outcomes is important because transient improvements during active treatment may not necessarily translate into sustained benefits during longer-term survivorship. Moreover, the available studies do not allow the effects of ketosis or carbohydrate restriction to be clearly separated from those of total energy intake, weight loss, physical activity, treatment-related symptoms, or psychological adaptation.

#### 6.5.2. Influence of Energy Intake and Nutritional Status

An additional consideration is that ketogenic diet may reduce appetite and spontaneous energy intake. In the study by Khodabakhshi et al. [27], caloric intake decreased by approximately 500 kcal/day during the intervention. Although this reduction may contribute to weight loss and metabolic changes, it represents an important confounder when QoL is used as an outcome. Reduced energy intake may have beneficial effects in patients with obesity and metabolic dysfunction, but may conversely increase the risk of fatigue, inadequate protein intake, loss of lean body mass and nutritional deterioration in patients who are already malnourished.

Therefore, QoL outcomes observed during ketogenic diet should be interpreted in conjunction with changes in body weight, energy and protein intake, body composition and nutritional status. In oncology patients, preservation of skeletal muscle and prevention of malnutrition remain fundamental objectives of nutritional care. Consequently, a reduction in caloric intake should not automatically be interpreted as a beneficial component of ketogenic diet.

#### 6.5.3. Overall Interpretation

Current evidence does not establish that ketogenic diet produces a sustained, clinically meaningful improvement in QoL among breast cancer patients. Some studies suggest possible short-term improvements in selected QoL domains, fatigue or emotional functioning [27,43], whereas other studies demonstrate no statistically significant improvement [78]. Importantly, methodological limitations—including lack of blinding, non-randomized dietary assignment, self-selection, small sample sizes, differential gastrointestinal symptoms between study groups, heterogeneous cancer treatments, and insufficient long-term follow-up—substantially limit causal interpretation [27,43,78,79].

Accordingly, ketogenic diet should currently be regarded as a potentially feasible supportive nutritional intervention rather than an established strategy for improving QoL in breast cancer patients. Future randomized controlled trials should use adequately powered samples, standardized ketogenic protocols, longer follow-up, and validated QoL instruments, while controlling for total energy and protein intake, weight change, physical activity, anticancer treatment and baseline nutritional status. Importantly, studies should distinguish the effects of ketogenic carbohydrate restriction from those of energy restriction and weight loss.

### 6.6. The Impact of the Ketogenic Diet on Body Composition and Metabolic Parameters in Oncology Patients, with a Focus on Breast Cancer

The study included 60 patients with metastatic breast cancer, who were divided into two groups. The intervention group (*n* = 30) followed a ketogenic diet supplemented with MCTs, while the control group (*n* = 30) followed a standard diet. The experiment was designed as a randomized clinical trial and lasted for 3 months. During the final follow-up visit, the authors observed a significant decrease in fasting blood glucose among the oncology patients following the MCT ketogenic diet. Additionally, an increase in blood ketone body concentration was observed, whereas this parameter did not change significantly in the control group. Furthermore, a statistically significant decrease in body weight, body fat, and BMI was noted in the intervention group. The authors demonstrated that the use of a high-fat diet did not negatively affect blood creatinine levels, provided that triglyceride concentrations remained normal. No significant changes were observed in the lipid profile. Despite the promising results, it must be noted that the study had a short duration, a small sample size, and did not specify the biological subtypes of breast cancer [27].

A reduction in body fat, body weight, and a decrease in BMI are beneficial, but exclusively in oncology patients struggling with obesity or overweight, because a weight gain of more than 10% following a breast cancer diagnosis is associated with an increased risk of mortality compared to patients who maintain a stable body weight [80].

In turn, a meta-analysis and systematic review by Taftian et al. [81] discussed the impact of the ketogenic diet on weight loss in oncology patients. Patient body weight is a significant prognostic factor in hormone-dependent breast cancers. The authors observed that weight loss was statistically significant in breast cancer patients following a ketogenic diet. In contrast, in feeding trials, no significant weight loss was observed in oncology patients. The authors note that the application of a ketogenic diet resulted in a statistically significant weight reduction only in the case of breast cancer patients.

Overweight in the HR+/HER2- (or HR+/HER2+) breast cancer subtype was associated with a lower overall survival (OS) rate, whereas obesity was associated with lower OS rates across all breast cancer subtypes [82].

Zhao et al. [40] analyzed the impact of the ketogenic diet on metabolic parameters and body composition in oncology patients. The authors included 10 trials representing 17 scientific articles in their meta-analysis. They concluded that the ketogenic diet can have a moderate to significant impact on the body weight of oncology patients. A decrease in BMI was observed in oncology patients following a ketogenic diet; however, only one study was analyzed for this parameter. The researchers suggest that the ketogenic diet may potentially contribute to a reduction in body fat. The ketogenic diet may have a minor impact on blood HDL and insulin concentrations. No statistically significant effect of ketogenic diets was demonstrated on TC, IGF-1, LDL, Cr, blood urea nitrogen (BUN), ALT, albumin, or TNF-α. However, a significant effect of the ketogenic diet on blood TG concentration was shown. The authors indicated that a ketogenic diet nutritional intervention could potentially reduce blood levels of free triiodothyronine (fT3) and gamma-glutamyl transferase (GGT). The authors demonstrated a statistically significant impact of the ketogenic diet on blood ketone body concentrations.

Amanollahi et al. [83] evaluated the impact of ketogenic diets on cardiometabolic outcomes in oncology patients. The researchers ultimately analyzed a total of 18 studies. Statistically significant decreases in body weight, BMI, and body fat were observed. The ketogenic diet significantly lowered FBG and IGF-1R. It was shown that the ketogenic diet did not have a significant impact on insulin concentration and C-reactive protein (CRP), but the authors note that decreases in insulin concentration were greater in breast cancer patient cohorts and in cohorts of patients under 60 years of age. No significant changes were demonstrated in TC, HDL, and LDL; however, highly statistically significant decreases in TG concentration were shown in breast cancer patients, patients under 60 years of age, and in cases of ketogenic diet nutritional interventions lasting less than 12 weeks. The authors demonstrated neither a positive nor a negative effect of the ketogenic diet on BUN, Cr, AST, or ALT indices. The ketogenic diet had a positive impact on QoL, but it was not statistically significant, with the exception of an improvement in emotional state.

Salido-Bueno et al. [84] observed only a statistically significant decrease in blood glucose concentration in oncology patients following a ketogenic diet. No statistically significant changes were noted regarding IGF-1 or the QoL index. No negative impact of the ketogenic diet on blood LDL concentration was observed. The authors did not demonstrate an effect of the ketogenic diet on weight loss.

### 6.7. Practical Monitoring and Implementation of Ketogenic Diets in Breast Cancer

Given the limited clinical evidence and the heterogeneous ketogenic protocols used in breast cancer studies, a standardized breast-cancer-specific ketogenic diet monitoring protocol has not yet been established. Nevertheless, an evidence-informed clinical framework may help reduce nutritional and metabolic risks in patients who elect to use ketogenic diet as a supportive intervention. Before initiating ketogenic diet, patients should undergo assessment of nutritional status, recent weight change, dietary intake, physical activity, treatment-related symptoms and planned anticancer therapy. Baseline laboratory assessment should include glucose and/or HbA1c, renal and hepatic function, electrolytes, magnesium, calcium and phosphate, with additional assessment of vitamin D, vitamin B12, folate and iron status according to individual risk. Cardiovascular assessment should include blood pressure and lipid profile, particularly in patients with pre-existing cardiovascular risk or receiving potentially cardiotoxic or metabolically active therapies. During the initial 2–4 weeks, follow-up should be more frequent, with assessment of body weight, dietary intake, gastrointestinal tolerance, hydration, treatment-related symptoms and relevant biochemical parameters. Subsequent monitoring should be individualized according to nutritional status, anticancer treatment and clinical stability. In patients at risk of malnutrition or sarcopenia, preservation of energy and protein intake should take priority over strict carbohydrate restriction.

There are currently no validated blood β-hydroxybutyrate or glucose targets specifically for breast cancer patients receiving ketogenic diet. Nutritional ketosis is commonly defined as β-hydroxybutyrate ≥ 0.5 mmol/L in ketogenic-diet research [74,85]; however, this threshold should not be interpreted as a breast-cancer-specific therapeutic target. Increasing ketone concentrations beyond this level has not been demonstrated to improve oncological outcomes. Therefore, treatment tolerance, nutritional adequacy and preservation of lean body mass should be prioritized over maximization of ketosis.

Different ketogenic approaches, including classical ketogenic diet, MCT-based ketogenic diet, modified Atkins diet and low-glycemic-index approaches, differ substantially in carbohydrate restriction, fat requirements, gastrointestinal tolerability and practical burden. Currently, there is insufficient evidence to establish that any particular variant is superior for patients receiving chemotherapy or radiotherapy. From a practical perspective, less restrictive approaches may be preferable in selected patients when they provide adequate nutritional intake and are better tolerated. MCT supplementation may facilitate ketone production but may also cause gastrointestinal symptoms and therefore should be introduced cautiously.

Importantly, adherence should be considered a clinical outcome in its own right. The feasibility of ketogenic diet may be limited by food cost, availability of suitable foods, preparation requirements, social restrictions, treatment-related nausea and altered taste, fatigue, gastrointestinal symptoms and the psychological burden of maintaining a highly restrictive dietary pattern during cancer treatment. Dietary counseling should therefore prioritize affordable, culturally appropriate and locally available foods rather than dependence on specialized ketogenic products.

Table 5 shows a practical monitoring framework for ketogenic diets in breast cancer patients.

Ketogenic diet should be modified or discontinued if clinically relevant nutritional deterioration, persistent gastrointestinal intolerance, dehydration, significant electrolyte abnormalities, symptomatic hypoglycemia, uncontrolled dyslipidemia, or inability to maintain adequate energy/protein intake occurs.

There are currently no validated breast-cancer-specific therapeutic targets for blood β-hydroxybutyrate or glucose during ketogenic dietary interventions. Nutritional ketosis should not be pursued at the expense of adequate energy and protein intake, particularly in patients at risk of cancer-related malnutrition or cachexia.

## 7. Conclusions

In this study, the available scientific evidence regarding the impact of the ketogenic diet on body composition, metabolic parameters, and quality of life in oncology patients, with a particular focus on women with breast cancer, was analyzed. Initial assumptions, based on the Warburg hypothesis, suggested that restricting glucose intake could inhibit the growth of cancer cells by limiting their primary energy source. However, current research indicates that tumor cells exhibit significant metabolic plasticity and are capable of utilizing alternative energy substrates, such as fatty acids, amino acids, or acetate.

To date, results from preclinical studies suggest potential benefits of implementing a ketogenic diet, including improvements in metabolic parameters, a possible delay in tumor progression, and an enhancement of the effects of selected targeted therapies. At the same time, clinical trial results remain inconclusive and do not allow for the confirmation of the ketogenic diet′s efficacy as a component of oncological treatment. Available studies are characterized by numerous methodological limitations, such as small sample sizes, high heterogeneity of the studied cohorts, short observation periods, and the risk of statistical bias.

An important aspect also remains the safety of the ketogenic diet. The reported adverse effects, including nausea, vomiting, and diarrhea, may deteriorate the nutritional status and treatment tolerance in oncology patients.

Ketogenic diet could lead to reduction in body fat rather than muscle mass. However, impact ketogenic diet on metabolic parameters is highly variable. On the one hand, ketogenic diet could reduce TG and IGF-1, but the current data is diverse, while on the other hand, ketogenic diet can lead to increases in TC, LDL-C, and ApoB-100. KD could lead to improvements in QoL and mental health in cancer patients, but current data is inconsistent.

Based on the analyzed literature, it cannot be unequivocally stated that the ketogenic diet exerts a beneficial or detrimental effect on body composition, metabolic parameters, or the mental health of oncology patients, including women with breast cancer. The current state of knowledge does not provide sufficient evidence to recommend the ketogenic diet as a safe and effective adjuvant method in cancer treatment. Further well-designed clinical trials with larger sample sizes and longer observation periods are necessary to unequivocally assess its efficacy and safety. We now clearly state that appropriately powered randomized controlled trials with clinically relevant endpoints are necessary before therapeutic conclusions can be drawn.

## Figures and Tables

**Table 1:** Summary of the evidence on the potential application of the ketogenic diet in oncology.

Reference	Type of Evidence	Population/Model	Main Findings	Conclusions
Zhao et al. [40]	Clinical study	Patients with breast, ovarian, and pancreatic cancer	↓ Body weight and body fat; preservation of muscle mass; no significant changes in selected renal and hepatic function parameters	KD may favorably affect body composition without clear deterioration of selected safety parameters
Kamali et al. [41]	Review of clinical studies	Patients with solid tumors	↓ Body weight, body fat, blood glucose, triglycerides, and IGF-1; preservation of lean body mass	KD may exert beneficial metabolic effects, although responses are heterogeneous among patients
Römer et al. [42]	Review of clinical evidence	Cancer patients	No conclusive evidence supporting a therapeutic anticancer effect of KD	KD should not currently be routinely used as an anticancer treatment
Kamali et al. [41]	Review of clinical studies	Patients undergoing chemotherapy and radiotherapy	Potential improvement in treatment tolerance and reduction of treatment-related adverse effects	KD may have a supportive role during conventional cancer treatment, but further clinical evidence is required
Li et al. [44]	Meta-analysis of preclinical studies	Rats and mice with various tumor types	↓ Tumor volume; ↑ survival; enhanced responses to chemotherapy, radiotherapy, and selected targeted therapies	KD shows promising anticancer effects in preclinical models and may have potential as an adjunctive treatment
Morscher et al. [45]	Preclinical studies	Neuroblastoma models	↓ Tumor progression, blood glucose, Ki-67, and pHH3 expression	Metabolic effects of KD may inhibit tumor cell proliferation in glucose-dependent tumors
Morscher et al. [45]	Preclinical study	SK-N-BE(2) neuroblastoma model with TP53 mutation and NMYC amplification	Lack of response to KD; increased utilization of glutamine as an alternative energy substrate	The efficacy of KD may depend on the genetic and metabolic characteristics of the tumor

Abbreviations: KD—ketogenic diet; IGF-1—insulin-like growth factor 1; Ki-67 and pHH3—markers of cellular proliferation; TP53—tumor protein p53; NMYC—MYCN oncogene; ↑—increase; ↓—decrease.

**Table 2:** Main methodological and clinical limitations of ketogenic diet studies in oncology.

Limitation	Description	Potential Impact on Interpretation
Caloric restriction as a confounding factor	Benefits attributed to ketogenic diet may partly result from reduced total energy intake rather than carbohydrate restriction or ketosis itself [46]	Makes it difficult to determine the independent effect of KD
Small sample sizes	Many studies include relatively few participants [5,47]	Reduces statistical power and limits generalizability
Short study duration	Interventions and follow-up periods are often relatively short [5]	Prevents assessment of long-term efficacy, safety, and adherence
Insufficient randomization	Some oncology studies have methodological shortcomings related to randomization [47]	Increases the risk of selection bias and confounding
Intensive nutritional counseling	Participants may receive substantial dietary counseling and clinical support in addition to the KD [47]	Makes it difficult to attribute observed effects specifically to KD
High dropout rates/poor adherence	The restrictive nature of KD and treatment-related factors may lead to discontinuation [47]	May introduce attrition bias and reduce the feasibility of KD in clinical practice
Adverse effects	Nausea, fatigue, gastrointestinal disturbances, and hypoglycemia have been reported [47]	May negatively affect adherence, quality of life, and treatment feasibility
Lack of standardized protocols	Studies differ in macronutrient composition, caloric intake, duration, and target ketone levels [48]	Limits comparability between studies and reproducibility of findings
Protocols not tailored to oncology	Some studies use KD protocols originally developed for epilepsy or obesity rather than cancer-specific protocols [48]	May not account for tumor type, treatment modality, or metabolic characteristics of cancer patients
Monitoring burden	Frequent measurement of blood glucose and ketone levels may be required [48]	Increases patient burden and may negatively affect adherence

Abbreviations: KD—ketogenic diet.

**Table 3:** Side effects and metabolic risks associated with the ketogenic diet.

Risk/Limitation	Evidence	Main Findings	Potential Relevance to Oncology
Alterations in lipid profile	Choi et al. [49]	↑ Total cholesterol and LDL-C in overweight/obese individuals without type 2 diabetes	May increase cardiovascular risk in susceptible patients
Changes in LDL particle characteristics	Choi et al. [49]; Burén et al. [50]	Changes in LDL particle distribution; Burén et al. reported ↑ small, dense LDL particles	May be relevant in patients with pre-existing cardiovascular risk
Increased ApoB-100	Burén et al. [50]	Significant ↑ in LDL-C and ApoB-100 following a controlled LCHF diet	Potential marker of increased atherogenic particle burden
Interaction with anticancer therapy	Yoo et al. [51]; Boszkiewicz et al. [52]	Aromatase inhibitors may adversely affect lipid parameters; anthracyclines have established cardiotoxic effects	Cardiovascular risks should be considered when KD is used alongside cardiotoxic or lipid-altering therapies
Gastrointestinal adverse effects	Schopf et al. [53]	Constipation, nausea, vomiting, and diarrhea frequently reported	May impair nutritional intake, quality of life, and adherence
Metabolic complications	Schopf et al. [53]	Hyperuricemia, metabolic acidosis, hypoglycemia, dehydration, and electrolyte disturbances reported	Potentially clinically significant in patients undergoing intensive cancer treatment
Neurological symptoms	Schopf et al. [53]	Lethargy and anorexia reported, particularly in pediatric populations	May further compromise nutritional status and treatment tolerance
Poor adherence/treatment discontinuation	Schopf et al. [53]	Restrictive dietary pattern and adverse effects may contribute to high dropout rates	Limits feasibility and may introduce attrition bias in clinical studies
Micronutrient deficiencies	Crosby et al. [7]; Engel et al. [54]	Potential deficiencies of folate, vitamins B1, B6, B12, A, E, K, calcium, magnesium, potassium, and iron	Particularly concerning in patients already at risk of malnutrition or treatment-related deficiencies
Insufficient dietary fiber	Engel et al. [54]; Crosby et al. [7]	Restriction of whole grains, fruits, and some vegetables may reduce fiber intake	May contribute to gastrointestinal problems and inadequate nutritional quality
Heterogeneity of dietary protocols	Crosby et al. [7]; Engel et al. [54]	Nutritional adequacy varies between classic KD, modified protocols, and contemporary low-carbohydrate approaches	Risks cannot be generalized to all forms of KD
Risk of nutritional deterioration	Crosby et al. [7]; Schopf et al. [53]; Engel et al. [54]	Restrictive intake may exacerbate existing nutritional deficiencies	Particularly important in patients with cancer, malnutrition, weight loss, or sarcopenia
Need for clinical monitoring	Crosby et al. [7]; Schopf et al. [53]	Monitoring of nutritional status, glucose, ketones, electrolytes, and hydration may be required	KD should be individualized and supervised by qualified healthcare professionals

Abbreviations: KD—ketogenic diet; LDL-C—low-density lipoprotein cholesterol; TC—total cholesterol; ApoB-100—apolipoprotein B-100; LCHF—low-carbohydrate high-fat diet; ↑—increase.

**Table 4:** Metabolic differences between breast cancer subtypes [57,64].

Breast Cancer Subtypes	Receptor Profile	Metabolic Profile	Main Metabolic Changes	Biological Significance
Luminal A	ER+, PR+, HER2−	More balanced metabolism, greater mitochondrial activity	↑ oxidative phosphorylation (OXPHOS), increased fatty acid utilization, lower glycolysis	Slower growth, usually less aggressive
Luminal B	ER+, often PR− and/or HER2+	More active metabolism than Luminal A	↑ glycolysis, ↑ glucose uptake (GLUT1), ↑ protein and lipid synthesis	Faster tumor growth, higher proliferative activity
HER2+	HER2+, ER/PR ±	Strongly reprogrammed energy metabolism	↑ glycolysis, ↑ GLUT1, ↑ lipid synthesis, ↑ nucleotide production	Rapid growth and higher biological aggressiveness
TNBC (Triple-Negative Breast Cancer)	ER−, PR−, HER2−	Most active and aggressive metabolic profile	↑ glycolysis (Warburg effect), ↑ glucose consumption, ↑ glutamine metabolism, ↑ oxidative stress, ↑ lipid synthesis	Fast growth, high invasiveness, more difficult treatment

Abbreviations: ER/PR—estrogen receptor/progesterone receptor; HER2—human epidermal growth factor receptor 2; GLUT1—glucose transporter 1; OXPHOS—oxidative phosphorylation; Warburg effect—preference for glycolysis despite the presence of oxygen; ↑—increase.

**Table 5:** Proposed pragmatic monitoring framework for ketogenic diets in breast cancer patients.

Domain	Baseline	First 2–4 Weeks	Long-Term Follow-Up
Weight/body composition	✓	every 1–2 weeks if high risk	individualized
Energy/protein intake	✓	✓	✓
GI symptoms/hydration	✓	✓	✓
Glucose/HbA1c	✓	according to risk	individualized
β-hydroxybutyrate	optional	optional	optional
Na/K/Mg/Ca/phosphate	✓	according to risk/symptoms	individualized
Renal/liver function	✓	according to treatment/risk	individualized
Lipid profile	✓	if clinically indicated	periodic
Vitamin D/B12/folate/iron	risk-based	risk-based	risk-based
Treatment tolerance	✓	✓	✓
Physical activity/function	✓	✓	✓

✓—indicates that parameters is assessed.

## Data Availability

No new data were created or analysed in this study. Data sharing is not applicable to this article.
